# Supplementary material for: Meta-Regression Analysis of Relationships between Fibre Type and Meat Quality in Beef and Pork—Focus on Pork
Source: Foods. 2023 May 31;12(11):2215. doi: 10.3390/foods12112215 (PMC10252544; doi:10.3390/foods12112215)
Supplement: Supplementary file 1 [file foods-12-02215-s001.zip › foods-2406705-supplementary.pdf]

Supplementary Materials:

Table S1: The studies analysed in the meta regression analysis including the authors, year, journal the work was published in, and the species associated with the study. Total number of animals used in the analysis (N), along with breed, sex of animals (M=male, F=female), and focus of the study.; information that was not provided is indicated by NA.

Author, Year	Year	Journal	Focus	Species	Breed	N	Sex
(Calkins, Dutson, Smith, Carpenter, & Davis)	1981	Journal of Food Science	Muscle fibre type and relation to marbling and tenderness of beef	Beef	NA	65	NA
(Seideman, Koohmaraie, & Crouse)	1987	Meat Science	Factors influencing beef tenderness	Beef	Hereford, Angus, Pinzgauer, Brahman, Saiwal	122	M
(Wegner et al.)	2000	Animal Science	How growth phases and breed impact muscle fibre characteristics in cattle; four breeds across seven different ages	Beef	German Angus, Galloway, Holstein Fresian, Belgian Blue	285	M
(Vestergaard et al.)	2000	Meat Science	Influence of feed on eating quality and muscle fibre characteristics from two seasons	Beef	Fresian	41	M
(Dransfield et al.)	2003	Meat Science	Quality and composition of different muscles from both cull cows and young bulls	Beef	Aubrac, Salers, Charlois, Limousin	168	M
(Jurie et al.)	2007	Meat Science	Impact of muscle characteristics of cull cows from different breeds	Beef	Holstein, Salers	13	F
(Hwang, Kim, Jeong, Hur, & Joo)	2010	Meat Science	Muscle fibre characteristics on marbling and quality of Hanwoo cattle	Beef	Hanwoo	18	NA
(Essén-Gustavsson & Fjelkner-Modig)	1985	Meat Science	The impact of muscle characteristics and different breeds on sensory properties	Pig	Hampshire Swedish Landrace Swedish Yorkshire	15	F

Author, Year	Year	Journal	Focus	Species	Breed	N	Sex
(Karlsson et al.)	1993	Journal of Animal Science	Histochemical properties of meat from lean pigs with varying diets and impact on quality	Pig	Swedish Yorkshire	82	M<br>F
(Maltin et al.)	1997	Meat Science	Investigation of the impact of fibre types on eating quality	Pig	NA	125	NA
(Henckel et al.)	1997	Meat Science	Investigation of muscle fibre type on performance in pork *longissimus* from different breeds and including gilts and castrated males	Pig	Danish Landrace<br>Danish Large White	300	M<br>F
(Huff et al.)	2002	Journal of Animal Science	Correlations of pork quality traits and muscle fibre type from 3 generations	Pig	Berkshire<br>Yorkshire	525	NA
(Chang et al.)	2003	Meat Science	Myosin heavy chain and impact on quality of traditional and modern pigs	Pig	Duroc<br>LargeWhite<br>Tamworth<br>Berkshire	192	M
(Fiedler, Nürnberg, Hardge, Nürnberg, & Ender)	2003	Meat Science	Muscle and fat variation in *longissimus* and impact on meat quality	Pig	Duroc x Berlin	245	NA
(Migdał et al.)	2004	Meat Science	Effect of dietary supplementation on meat and eating quality finished at different weights	Pig	Large White x Landrace x Pietrain	40	M
(Melody et al.)	2004	Journal of Animal Science	Biochemical factors postmortem and impact on tenderness and water holding capacity	Pig	Duroc	16	M

Author, Year	Year	Journal	Focus	Species	Breed	N	Sex
(Ryu & Kim)	2005	Meat Science	Muscle fibre characteristics on postmortem metabolism and meat quality	Pig	Duroc x Yorkshire x Landrace	231	M<br>F
(Gil et al.)	2008	Meat Science	Impact of genetics on fibre type , biochemical trains, and meat quality	Pig	Five genetic lines from PIC	399	F
(Shin et al.)	2008	Food Science and Biotechnology	Relationship of tenderness and quality traits to fibre type	Pig	NA	103	F
(Nam et al.)	2009	Meat Science	Relationship of quality traits, muscle fibre type, and sensory characteristics	Pig	Landrace x Yorkshire x Duroc	133	NA
(Lee, Choe, Choi et al.)	2012	Meat Science	Quality and muscle fibre characteristics from different breeds	Pig	Berkshire<br>Duroc<br>Landrace<br>Yorkshire	243	M<br>F
(Jeong et al.)	2010	Meat Science	Sensory properties of cooked pork and relationship to fatty acid composition, fibre type, and quality	Pig	Berkshire	113	M<br>F
(Kang et al.)	2011	Meat Science	Effect of fibre type on quality, fatty acid composition, and sensory properties	Pig	Berkshire	85	M<br>F
(Smith et al.)	2011	Journal of Animal Science	Effect of selection of feed intake on quality	Pig	Yorkshire	16	M
(Kim, Jeong, et al.)	2013	Meat Science	Impact of fibre type IIb on carcass and meat quality traits	Pig	Korena Native x Landrace	96	M<br>F
(Kim, Kim, et al.)	2013	Food Bioprocess Technology	Relationship of carcass weight and fibre type on quality	Pig	Korean Native Black x Landrace	168	NA
(Li et al.)	2014	Meat Science	Effect of ractopamine and castration methods on muscle	Pig	Pietrain	1488	NA

Author, Year	Year	Journal	Focus	Species	Breed	N	Sex
			fibre and sensory quality between genotypes				
(Kim, Lee, & Ryu)	2018	Animal Production Science	Meat quality and fibre type across breeds and sexes	Pig	Berkshire<br>Duroc<br>Landrace<br>Meishan<br>Yorkshire<br>Landrace x Yorkshire x Duroc	1374	M<br>F
(Velotto et al.)	2018	Italian Journal of Food Science	Investigation of relationship of fibre type, fatty acid composition, and sensory	Pig	Cinta Senese	65	M<br>F
(Lowell et al.)	2018	Journal of Animal Science	Correlations of fresh and aged *longissimus* quality between Duroc and Pietrain pigs	Pig	Duroc<br>Pietrain	320	M<br>F
(Kim, Overholt, et al.)	2018	Meat And Muscle Biology	How fibre characteristics impact muscle fibre volume and pork quality	Pig	NA	30	M<br>F
(Song et al.)	2020	Foods	Pork quality and fibre characteristics affected by direction of cut	Pig	Landrace x Yorkshire x Berkshire	15	NA
